# Phenolic–Bioactivity Connectivity Networks Reveal How Lactic Fermentation Restructures Function in Murta Berry Juice

**DOI:** 10.3390/antiox15070832

**Published:** 2026-07-01

**Authors:** Cristian J. Gomes-Lobo, Wendy Franco, Mario Faundez, Óscar Martínez-Álvarez, José R. Pérez-Correa

**Affiliations:** 1 Chemical and Bioprocess Engineering Department, School of Engineering, Pontificia Universidad Católica de Chile, Vicuña Mackenna 4860, Santiago 7820436, Chile; cjgomes@uc.cl (C.J.G.-L.); wfranco@uc.cl (W.F.); 2 Research and Innovation Center VitiScience, ANID CIA 250013, Santiago 7820436, Chile; 3 Department of Agroindustrial Engineering, El Paraíso Campus, National Autonomous University of Honduras (UNAH), Danlí 13201, Honduras; 4 Pharmacy Department, Faculty of Chemistry, Pontificia Universidad Católica de Chile, Vicuña Mackenna 4860, Santiago 7820436, Chile; mfaundeza@uc.cl; 5 Institute of Food Science, Technology and Nutrition (ICTAN, CSIC), 6 José Antonio Novais St, 28040 Madrid, Spain; oscar.martinez@ictan.csic.es

**Keywords:** lactic acid bacteria, phenolic–bioactivity connectivity, polyphenol fractions, enzyme inhibition, oxidative stress, *Ugni molinae*

## Abstract

Lactic fermentation modulates polyphenol composition in plant matrices, yet how compositional shifts translate into functional outcomes across extractable (EP) and hydrolyzable (HP) fractions remains unclear. Here, we apply a phenolic–bioactivity connectivity framework to fermented murta (*Ugni molinae* Turcz) juice, integrating compositional profiling with three functional axes: antimicrobial activity (against *Escherichia coli*, *Salmonella enterica*, and *Staphylococcus aureus*), inhibition of carbohydrate-hydrolyzing enzymes (α-amylase, α-glucosidase) and DPP-IV, and modulation of oxidative stress in Caco-2 cells. Murta juice was fermented with *Lactobacillus acidophilus*, *Lactiplantibacillus plantarum*, and a 1:1 coculture under two optimized strategies (GDF and SAW). Principal component analysis separated fermented from unfermented samples (89.7% variance explained) and identified coculture fermentation (MIX-GDF) as the most compositionally distinct treatment. EP fractions drove antimicrobial and α-glucosidase inhibition, whereas HP fractions contributed preferentially to DPP-IV inhibition and intracellular reactive oxygen species (ROS). A bipartite correlation network revealed a dual-functional architecture: specific flavonoid–bioactivity associations governed enzyme inhibition, while diffuse collective interactions shaped antimicrobial responses. These results demonstrate that fermentation-induced phenolic remodeling yields structured, functional outcomes, providing a rational basis for designing fermentation strategies targeting specific bioactivity profiles.

## 1. Introduction

Polyphenols determine much of the biological functionality of plant-based foods, yet their bioactivity depends on their distribution between extractable (EP) and non-extractable fractions, including hydrolyzable polyphenols (HP). HP remains underexplored despite representing a substantial share of total phenolics [1,2]. Their functional relevance increases once released under physiological conditions [3].

Lactic acid fermentation (LAF) is an effective strategy for modulating the availability and functionality of phenolic fractions, promoting the release of bound compounds, facilitating their breakdown into lower-molecular-weight metabolites, and improving bioavailability [1]. Lactic acid bacteria (LAB), including *Lactiplantibacillus plantarum* and *Lactobacillus acidophilus*, transform phenolic profiles through enzymatic mechanisms [4,5]. However, most studies report individual bioactivities (antimicrobial, antioxidant, or enzyme-inhibitory) in isolation, without establishing quantitative links between compositional changes and the resulting functional landscape.

*Ugni molinae* Turcz., known locally as murta or murtilla, is a berry-producing shrub indigenous to southern Chile and Argentina. Its fruits are characterized by a pleasant fruity aroma and a balanced sweet–tart taste, making them suitable for both fresh consumption and processing into a range of food products. The fruit is characterized by a high-water content (~77%), soluble solids ranging from 14 to 17 °Brix in cultivated varieties and higher values in some wild ecotypes, together with carbohydrates (17–18%), dietary fiber (~2.7%), and vitamin C. Additionally, it contains a complex polyphenolic profile [6], including phenolic acids, anthocyanins, and flavonols. The berries are rich in myricetin and quercetin derivatives that contribute to their characteristic pigmentation and have been associated with antioxidant, antimicrobial, and anti-inflammatory properties [7,8]. However, preserving the bioactive quality of murta juice remains a technological challenge because phenolic compounds, particularly anthocyanins, can undergo oxidation and degradation reactions during processing and storage. Recent work has shown that LAF is an effective strategy for modulating EP and HP compositions in murta juice, promoting phenolic transformation and improving antioxidant potential under optimized fermentation conditions [9,10].

Yet no study has simultaneously evaluated the biological functionality of both fractions across multiple bioactivity axes following controlled fermentation, nor has it attempted to link compositional shifts to functional outcomes through integrative analysis.

This study addresses that gap by applying a phenolic–bioactivity connectivity framework to fermented murta juice. We characterize the antimicrobial, antidiabetic (α-amylase, α-glucosidase, DPP-IV), and cytoprotective (Caco-2 cell viability and intracellular ROS) responses of EP and HP fractions obtained under monoculture and coculture fermentation conditions. By integrating principal component analysis with bipartite correlation networks, we identify both specific phenolic–bioactivity associations and collective interaction patterns, revealing how fermentation restructures the functional architecture of polyphenol-rich juice.

## 2. Materials and Methods

### 2.1. Chemicals and Reagents

All chemicals and reagents used were of analytical or high-performance liquid chromatography (HPLC) grade. Mueller-Hinton Broth (MHB) was obtained from Condalab (Madrid, Spain). Nitrogen (N_2_) was obtained from Air Liquide (Madrid, Spain). Potassium monophosphate (KH_2_PO_4_), potassium diphosphate (K_2_HPO_4_), starch, and potassium sodium tartrate tetrahydrate (C_4_H_4_H_4_KNaO_6_-4H_2_O) reagents were obtained from Merck (Darmstadt, Germany). Porcine pancreatic α-amylase, 3,5-dinitrosalicylic acid (DNS), sodium hydroxide (NaOH), α-glucosidase enzyme, and 4-nitrophenyl-α-D-glucopyranoside (PNPG) substrate were obtained from Sigma-Aldrich (Saint Louis, MO, USA). Dipeptidyl peptidase IV (DPP-IV, EC 3.4.14.5) from porcine kidney, hippuril-His-Leu, angiotensin II, vitamin B_12_, aprotinin, Hank’s Balanced Salt Solution (HBSS), and Phosphate-Buffered Saline (PBS) buffer solutions were provided by Sigma-Aldrich, Co. (St. Louis, MO, USA). The chromogenic substrate (H-Gly-Pro-AMC-HBr) was obtained from Bachem (Bubendorf, Switzerland). Dulbecco’s Modified Eagle Medium (DMEM) was obtained from Biological Industries (Kibbutz Beit Haemek, Israel). The reagent DCFH-DA (carboxy-2′,7′-dichlorodihydrofluorescein diacetate) and dimethyl sulfoxide (DMSO) were acquired from Sigma (Madrid, Spain). Human colorectal adenocarcinoma Caco-2 cells were obtained from American Type Culture Collection, Manassas, VA, USA. The antibiotics penicillin (10,000 U/mL) and streptomycin (10,000 μg/mL), fetal bovine serum (FBS), and 2.5% trypsin/EDTA solution (10×) were provided by Cytiva/HyClone (Marlborough, MA, USA). The Alamar Blue reagent was obtained from Thermo Scientific (Waltham, MA, USA), and cell culture plates and flasks were obtained from Sarstedt (Barcelona, Spain).

### 2.2. Samples

Red murta berries (*Ugni molinae* Turcz) were harvested in April 2023 in Coihuería, Osorno (40°31′00″ S, 73°24′00″ W), Los Lagos Region, southern Chile, and stored at −20 °C until processing. Six fermentation conditions were evaluated (Table 1), combining two optimized strategies: the Global Desirability Function (GDF) strategy characterized by high temperatures and high inoculum, and the Simple Additive Weighting (SAW) strategy characterized by low temperatures and low inoculum [10]. These were applied to three cultures: *Lactobacillus acidophilus* LA-05 (La), *Lactiplantibacillus plantarum* LM PRIME (Lp), and a 1:1 mixed culture (MIX). The fermented samples were freeze-dried, and extraction was performed on a dry weight basis, after which EP was obtained by sequential solvent extraction using MeOH/H_2_O (50:50, *v*/*v*) followed by acetone/H_2_O (70:30, *v*/*v*) at a sample-to-solvent ratio of 1:10 (*w*/*v*). Each extraction step was performed at room temperature for 1 h under agitation, followed by centrifugation for 10 min. HP was obtained from the residues by acid hydrolysis (MeOH + H_2_SO_4_, 85 °C, 20 h), followed by purification by solid-phase extraction (SPE) with Oasis HLB cartridges (5400 mg, 3 cm^3^, 30 μm; Waters, Milford, MA, USA) [10]. For each assay, the required volume of each extract was dried under a flow of ultrapure nitrogen (N_2_), and the resulting residues were resuspended in sterile PBS to obtain the corresponding stock solutions. Different concentrations of each extract and fraction were subsequently prepared according to the requirements of each assay.

### 2.3. Bacteria Culture Preparation

For the antibacterial activity assay, three model food pathogenic bacterial strains were used: *Escherichia coli* ATCC^®^ 35218™ (*E. coli*), *Staphylococcus aureus* ATCC^®^ BAA-1026™ (*St. aureus*), and *Salmonella enterica* ATCC^®^ 49223™ (*S. enterica*). The strains were purchased in KWIK-STIK^®^ format (Microbiologics^®^, Saint Cloud, MN, USA). Strains were cultured in Mueller-Hinton broth and incubated at 37 ± 2 °C for 24 h in an orbital shaker (SI500; Richmond Scientific, Chorley, UK).

#### Microbial Inhibition Kinetics

The antimicrobial activity of EP and HP extracts was assessed against *Escherichia coli* (*E. coli*), *Staphylococcus aureus* (*S. aureus*), and *Salmonella enterica (S. enterica*), using the microdilution assay in 96-well microplates [11]. Each well contained 40 μL of the samples diluted in PBS, 10 μL of bacterial culture adjusted to 10^6^ CFU/mL, and 50 μL of MHB. Five final concentrations were tested for the EP fraction (200, 400, 600, 800, and 1000 µg/mL) and for the HP fraction (1200, 1400, 1600, 1800, and 2000 µg/mL). Bacterial growth was monitored every hour for 24 h at 630 nm using a TECAN Infinite^®^ M200 Pro microplate reader (Tecan Group, Männedorf, Switzerland), with a 10 s shaking step (5 mm amplitude) before each measurement. Inhibitory activities were expressed as inhibition percentage (%) according to the following equation:

(1) $$\%\;Activity=\frac{{Abs}_{control}-{Abs}_{sample}}{{Abs}_{control}}\;\times \;100$$

where *Abs_control_* is the absorbance at 630 nm of the negative control (bacterial cultures in MHB without extract), and *Abs_sample_* is the absorbance of wells containing extract.

### 2.4. Inhibitory Activity Against Metabolic Syndrome-Related Enzymes

#### 2.4.1. α-Amylase Inhibitory Activity

The inhibitory activity of the extracts against α-amylase was measured using the chromogenic substrate assay described by [12], with some modifications. A potassium phosphate buffer (20 mM, containing 5 mM NaCl, pH 6.8) was used together with an α-amylase solution from porcine pancreas (0.5 mg/mL) and starch (0.5% *w*/*v*) as the substrate. Samples were diluted to final concentrations of 200–1000 μg/mL for the EP fraction and 1200–2000 μg/mL for the HP fraction. Enzymatic reactions were carried out in Eppendorf tubes by sequentially adding the substrate and enzyme, then adding 200 μL of DNS reagent. The reaction mixtures were then heated at 100 °C for 5 min, cooled to room temperature, and 50 μL aliquots were transferred to 96-well microplates. Each aliquot was diluted with 200 μL of distilled water, and absorbance was measured at 540 nm using a microplate reader (Infinite^®^ M200 Pro, Tecan Group). Distilled water was used as the blank. The percentage of enzymatic inhibition was calculated according to Equation (1).

#### 2.4.2. α-Glucosidase Inhibitory Activity

α-Glucosidase inhibition was assayed using the chromogenic assay described by [13], with some modifications. A 100 mM sodium phosphate buffer (pH 6.9) was prepared and used to dissolve α-glucosidase (0.5 U/mL) and p-nitrophenyl-α-D-glucopyranoside (PNPG, 5 mM) as substrate. Samples were diluted to final concentrations of 200–1000 μg/mL for the EP fraction and 1200–2000 μg/mL for the HP fraction. In each well, 20 μL of sample and 70 μL of enzyme solution were added, and the mixture was pre-incubated at 37 °C for 5 min in a 96-well microplate reader (Infinite^®^ M200 Pro, Tecan Group). The reaction was initiated by adding 50 μL of PNPG solution. Absorbance was monitored at 405 nm every 3 min for 30 min at 37 °C. Blanks without enzyme and phosphate buffer were used as negative controls. The percentage of enzyme inhibition was calculated according to Equation (1).

#### 2.4.3. DPP-IV Inhibitory Activity

DPP-IV (dipeptidyl peptidase IV) inhibitory activity was assessed using H-Gly-Pro-4-nitroanilide acetate (Gly-Pro-p-NA) as a substrate, as described previously [14]. A 100 mM Tris–HCl buffer (pH 8.0) was prepared and used to dilute dipeptidyl peptidase IV (DPP-IV, 10.64 mU) and to prepare the fluorogenic substrate H-Gly-Pro-AMC-HBr (25 μM). Samples were dissolved in the same buffer to obtain a final concentration of 1 mg/mL per well. In each well, 20 μL of enzyme solution was combined with either 180 μL of working buffer (control) or 150 μL of working buffer plus 30 μL of sample, then incubated at 37 °C for 15 min in a 96-well microplate reader (Clariostar Plus, BMG Biotech, Ortenberg, Germany). The reaction was initiated by adding 100 μL of substrate solution. Fluorescence was recorded every minute for 15 min (excitation/emission: 340/440 nm). Inhibitory activity was calculated from the initial fluorescence slopes in the presence and absence of the samples, and the results were expressed as percentage inhibition.

### 2.5. Evaluation of Cell Viability and Oxidative Stress

#### 2.5.1. Cell Culture and Treatment

Human colorectal adenocarcinoma Caco-2 cells (ATCC ref. HTB-37) were obtained from the American Type Culture Collection (ATCC, Manassas, VA, USA). Cells at passages 12–15 were cultured in DMEM supplemented with 10% FBS, 1× nonessential amino acids solution, 100 U/mL penicillin, and 100 μg/mL streptomycin. The culture was maintained in a humidified incubator at 37 °C with a controlled atmosphere of 5% CO_2_.

#### 2.5.2. Cell Viability Assay

Cell viability was determined following the methodology described by [15,16]. Cells were seeded in 96-well flat-bottom plates at a density of 5 × 10^3^ cells per well in 100 μL and incubated for 24 h under standard culture conditions. EP and HP fractions were then added at final concentrations of 45–225 μg/mL, prepared in PBS with a maximum solvent content of 10% (*v*/*v*). In each well, cells were incubated with the corresponding sample for 72 h at 37 °C in a humidified atmosphere containing 5% CO_2_. Subsequently, 10 μL of Alamar Blue reagent was added to each well, and plates were further incubated for 5 h under the same conditions. Fluorescence was measured at excitation and emission wavelengths of 560 and 590 nm, respectively, using a microplate reader (BioTek Cytation 5, Agilent Technologies, Santa Clara, CA, USA). Results were expressed as percentage inhibition.

#### 2.5.3. Protective Effect Against H_2_O_2_-Induced Oxidative Stress

The protective effect of EP and HP fractions against H_2_O_2_-induced oxidative stress was evaluated as described in [17], with slight modifications. Caco-2 cells were seeded in 96-well flat-bottom culture plates at a density of 5 × 10^3^ cells per well in 100 μL of DMEM supplemented with 10% FBS. Oxidative stress was then induced by treatment with H_2_O_2_ at a final concentration of 20 μM for 24–48 h [18]. EP and HP fractions were subsequently added at final concentrations of 45–225 μg/mL, prepared in PBS. In each well, cells were treated with the corresponding sample under the same incubation conditions. Subsequently, 10 μL of Alamar Blue reagent was added to each well, and the plates were incubated for an additional 5 h. Fluorescence was measured at excitation and emission wavelengths of 560 and 590 nm, respectively, using a microplate reader (BioTek Cytation 5, Agilent Technologies).

#### 2.5.4. Intracellular Reactive Oxygen Species (ROS) Measurement

Intracellular reactive oxygen species were measured using the methodology described by [15] with some modifications. Caco-2 cells were seeded at a density of 4 × 10^4^ cells per well in 96-well fluorescence flat-bottom plates using sterile HBSS under standard culture conditions. Cells were then pre-incubated with 20 μM 2′,7′-dichlorofluorescin diacetate (DCFH-DA) at 37 °C for 30 min. In each well, cells were washed twice with sterile HBSS, then treated with EP and HP fractions in combination with H_2_O_2_ (final concentration: 20 μM). Fluorescence was recorded every 2 min for 60 min at excitation and emission wavelengths of 488 and 510 nm, respectively, using a microplate reader (BioTek Cytation 5, Agilent Technologies). Intracellular ROS levels were quantified as the ratio of the slope of fluorescence generation under each treatment to that of the control, and the results were expressed as percentage inhibition.

### 2.6. Statistical Analysis

All measurements were performed in triplicate and independently repeated in two separate experiments, yielding 6 replicate values. Analyses and graphical representations were conducted using GraphPad Prism (version 7.0.5). Multivariate analyses were carried out in Python 3.11.5. Phenolic data were standardized using the StandardScaler function, and principal component analysis (PCA) was performed using the scikit-learn PCA function. Data handling was conducted with pandas, and biplots of the first two principal components were generated using Matplotlib 3.7.2. The Mantel test was performed using the ‘mantel’ function from scikit-bio, with Euclidean distance matrices computed via ‘pdist’ and ‘squareform’ from SciPy. Significant phenolic–bioactivity associations were visualized as a bipartite network constructed with NetworkX and plotted using Matplotlib.

## 3. Results and Discussion

### 3.1. Fermentation Reshapes the Phenolic Landscape of EP and HP Fractions

**Figure 1:**
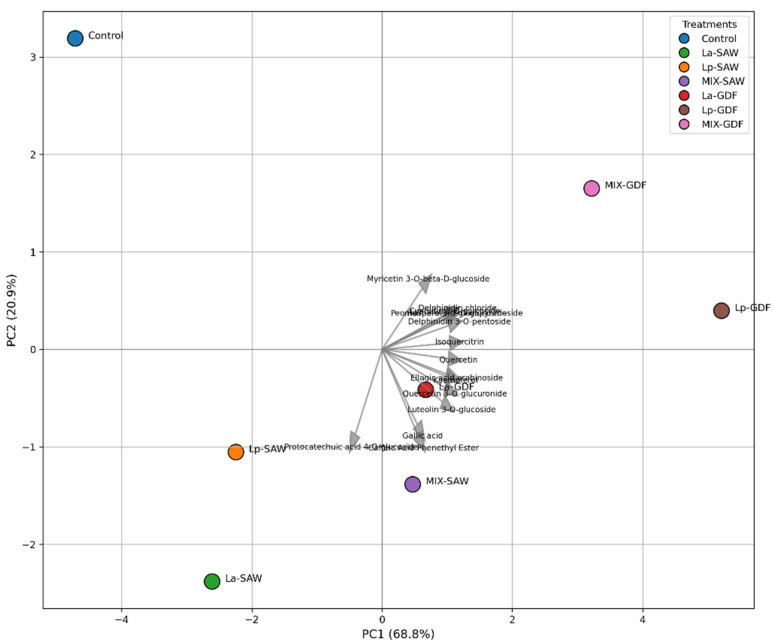
Biplot of principal component analysis (PCA) based on phenolic compounds quantified in murta juice fermented with different strains (*Lactobacillus acidophilus* (La), *Lactiplantibacillus plantarum* (Lp), and mixed culture (MIX)) and optimized fermentation conditions (Simple Additive Weighting (SAW) and Global Desirability Function (GDF)).

To establish the compositional framework underpinning all subsequent bioactivity comparisons, we first examined how fermentation conditions redistribute phenolic compounds across EP and HP fractions. Principal component analysis (PCA) of 15 phenolic compounds with high relative abundance (previously characterized by [10]) was used to summarize phenolic variation across fermentation conditions. As shown in Figure 1, the first two principal components explained 89.7% of the total variance (PC1: 68.8%; PC2: 20.9%), clearly separating fermented from unfermented samples and identifying coculture fermentation (MIX-GDF) as the most compositionally distinct treatment. PC1 was mainly driven by myricetin 3-O-β-D-glucoside, quercetin 3-O-glucuronide, luteolin 3-O-glucoside, and gallic acid, which clustered with MIX-GDF on the positive side of the axis. PC2 was more strongly influenced by protocatechuic acid 4-O-glucoside and caffeic acid phenethyl ester, which were more closely associated with SAW treatments. These loading patterns indicate that fermentation conditions shape the phenolic landscape and, consequently, the functional potential of the juice.

A Mantel test confirmed a statistically significant correlation between the phenolic composition distance matrix (Euclidean) and the bioactivity distance matrix (Euclidean) (r = 0.68, *p* = 0.04, 9999 permutations; see Section 3.5 for the full network analysis).

### 3.2. Antimicrobial Activity of EP and HP Fractions

**Figure 2:**
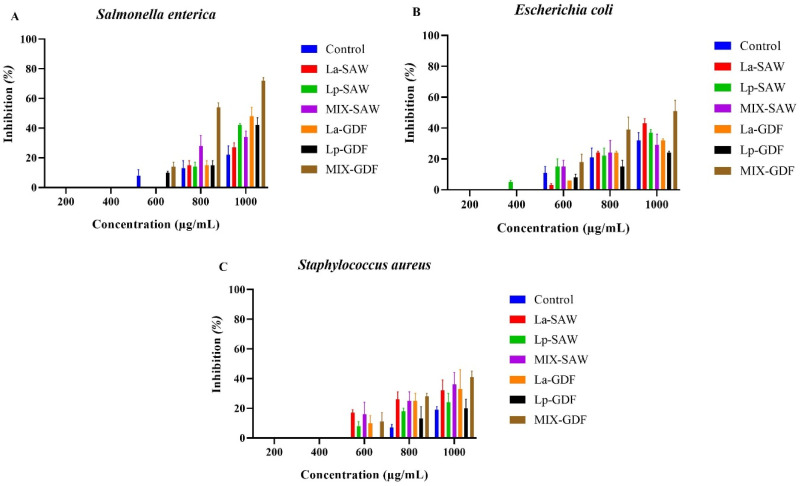
Inhibition (%) of *Salmonella enterica* (**A**), *Escherichia coli* (**B**), and *Staphylococcus aureus* (**C**) by the extractable phenolic (EP) fraction obtained from fermented murta juice. La: *Lactobacillus acidophilus*; Lp: *Lactiplantibacillus plantarum*; MIX: 1:1 mixed culture of both strains; SAW: Simple Additive Weighting; GDF: Global Desirability Function.

As shown in Figure 2, EP fractions exhibited a strong and dose-dependent antimicrobial activity (250–1000 µg/mL), with maximum inhibition under coculture fermentation conditions (MIX-GDF), reaching approximately 70% against *S. enterica*, 55% against *E. coli*, and 50% against *S. aureus* at 1000 µg/mL. This behavior is consistent with the predominance of low-molecular-weight, freely soluble phenolics, such as hydroxycinnamic acids and flavonoids, previously identified in the EP fraction. These compounds are known to exert antimicrobial effects primarily through interactions with bacterial membranes, where their amphiphilic nature enables insertion into lipid bilayers, thereby increasing permeability and leakage of intracellular constituents [19]. In addition, phenolic compounds can exert antibacterial activity by destabilizing cell membranes, increasing permeability, inhibiting microbial enzymes, and disrupting essential metabolic processes, thereby limiting bacterial growth [20]. The greater susceptibility of Gram-negative bacteria can be attributed to the diffusion of small phenolics through porin channels and their interaction with lipopolysaccharides in the outer membrane, thereby destabilizing the membrane structure [21,22].

The increase in activity observed under MIX-GDF conditions suggests that microbial interactions during co-fermentation promote enzymatic deglycosylation and deesterification, thereby generating more lipophilic aglycones with greater membrane affinity and higher biological activity. These transformations have been extensively described in fermented plant matrices, where microbial β-glucosidase and esterase activities increase the release of bioactive phenolic compounds, thereby enhancing their antimicrobial activity [23]. Increased lipophilicity has been directly correlated with improved antimicrobial efficacy due to enhanced partitioning into bacterial membranes and greater intracellular accessibility [24]. Moreover, phenolic compounds can induce oxidative stress in bacterial cells by generating reactive oxygen species or interfering with redox homeostasis, further contributing to antimicrobial activity [20].

**Figure 3:**
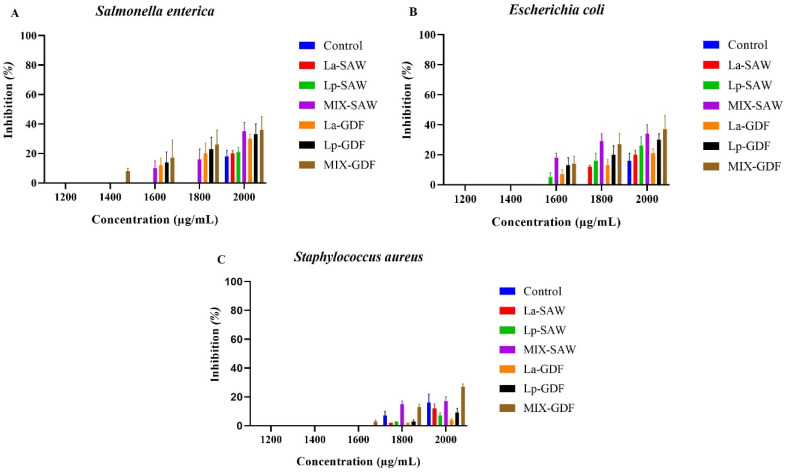
Inhibition (%) of *Salmonella enterica* (**A**), *Escherichia coli* (**B**), and *Staphylococcus aureus* (**C**) by the hydrolyzable phenolic (HP) fraction obtained from fermented murta juice. La: *Lactobacillus acidophilus*; Lp: *Lactiplantibacillus plantarum*; MIX: 1:1 mixed culture; SAW: Simple Additive Weighting; GDF: Global Desirability Function.

In contrast, HP fractions exhibited a more moderate antimicrobial activity, becoming evident only at higher concentrations (1200–2000 µg/mL), as shown in Figure 3. This reduced efficacy is consistent with the structural constraints of matrix-bound phenolics, which are often present as high-molecular-weight polymers or covalently linked to cell wall components, limiting their diffusivity and interactions with microbial targets [25]. However, fermentation can partially overcome these limitations through microbial enzymatic activity, promoting the release and transformation of bound phenolics into smaller, more bioactive molecules [23,26].

The increased activity of HP fractions under coculture conditions further supports the role of microbial consortia in enhancing enzymatic diversity and catalytic efficiency, facilitating reactions such as glycosidic cleavage and partial depolymerization. These processes increase the accessibility of phenolic compounds and may also generate derivatives with improved bioactivity. In addition, HP fractions may contain other matrix-associated compounds, including sterols and pentacyclic triterpenoids, which contribute to antimicrobial effects through complementary mechanisms such as membrane perturbation and enzyme inhibition [26].

These results indicate that lactic fermentation enhances antimicrobial functionality through the combined effects of phenolic release, structural transformation, and increased bioavailability. The EP fraction provides a rapid and potent antimicrobial response driven by diffusible, membrane-active compounds. In contrast, the HP fraction contributes a secondary, sustained effect as bound phenolics are progressively liberated and activated. HP contribution is less pronounced but biologically relevant within a complex food matrix. This complementary behavior explains the superior antimicrobial performance observed under coculture fermentation. It underscores the importance of considering both extractable and hydrolyzable phenolic pools when evaluating the antimicrobial potential of fermented plant-based systems. It should be noted that the HP fraction required substantially higher concentrations (1200–2000 μg/mL) than the EP fraction (200–1000 μg/mL) to produce comparable biological effects. While these concentrations exceed those typically achievable in the systemic circulation, they are relevant in the gastrointestinal lumen, where phenolic concentrations following a polyphenol-rich meal can reach the low-mg/mL range before absorption. Moreover, HP compounds are progressively released during colonic fermentation and can accumulate locally at the intestinal epithelium [2]. Therefore, the HP concentrations tested here are physiologically plausible in the gut environment, the primary site of action for dietary polyphenols before systemic absorption.

### 3.3. Antidiabetic Potential: Enzyme Inhibition by EP and HP Fractions

The inhibitory activity of EP and HP fractions from fermented and unfermented murta juice was assessed against three enzymes central to glycemic regulation: α-amylase, α-glucosidase, and DPP-IV. Results for each enzyme are presented next.

#### 3.3.1. α-Amylase Inhibition Activity

α-Amylase catalyzes the initial step of starch digestion by hydrolyzing α-(1→4) glycosidic bonds, producing oligosaccharides that are subsequently converted into absorbable monosaccharides in the small intestine, thereby contributing to postprandial glycemic excursions. Accordingly, partial inhibition of α-amylase has been widely recognized as an effective strategy to attenuate glucose release and modulate glycemic response, reducing the risk of type 2 diabetes and related metabolic disorders [27,28].

**Figure 4:**
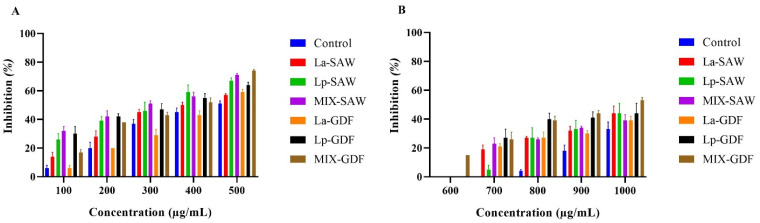
Enzymatic inhibition of α-amylase by phenolic extracts from fermented murta juice. (**A**) Extractable phenolic (EP) fraction; (**B**) hydrolyzable phenolic (HP) fraction. Results are expressed as percentage inhibition at different concentrations. La: *Lactobacillus acidophilus*; Lp: *Lactiplantibacillus plantarum*; MIX: 1:1 mixed culture; SAW: Simple Additive Weighting; GDF: Global Desirability Function.

The EP fraction exhibited a clear concentration-dependent inhibition of α-amylase, with fermented samples consistently showing higher inhibitory activity than the unfermented control (Figure 4A). Inhibition increased progressively with extract concentration across the entire range tested. Coculture-fermented extracts (MIX-SAW and MIX-GDF) displayed the strongest inhibition, reaching approximately 70–75% at the highest concentrations, whereas the unfermented control showed only minimal activity. This enhancement can be mechanistically attributed to fermentation-induced transformations of phenolic compounds, including enzymatic deglycosylation, de-esterification, and structural modification by microbial metabolism. These processes generate lower-molecular-weight phenolics and aglycones, increasing their accessibility to the enzyme active site and improving their binding affinity.

At the molecular level, polyphenols inhibit α-amylase primarily through non-covalent interactions, including hydrogen bonding and hydrophobic interactions with catalytic residues such as Asp197, Glu233, and Asp300, which are essential for substrate hydrolysis [29,30]. Inhibition strength is strongly influenced by structural features such as the number and position of hydroxyl groups, degree of polymerization, and glycosylation state. In particular, aglycone forms of flavonoids and phenolic acids exhibit enhanced inhibitory activity due to increased planarity and hydrophobicity, which favor interaction with the enzyme active site and stabilization of enzyme–inhibitor complexes [31]. These structure–activity relationships are consistent with the higher inhibitory capacity observed in coculture-fermented samples, where microbial enzymatic activity promotes the release of more active phenolic forms.

The HP fraction also showed concentration-dependent inhibition of α-amylase (Figure 4B), although the inhibitory effect was lower than that observed for the EP fraction. At higher concentrations (900–1000 µg/mL), coculture-fermented extracts (MIX-GDF and MIX-SAW) reached inhibition levels of approximately 50–60%, whereas the unfermented control remained substantially lower. This comparatively reduced activity can be explained by the structural characteristics of hydrolyzable polyphenols, which are often present as high-molecular-weight compounds or bound to the plant matrix, limiting their diffusivity and steric accessibility to the enzyme active site. However, fermentation partially mitigates these constraints by promoting enzymatic hydrolysis and depolymerization, thereby releasing smaller phenolic units that can interact with α-amylase.

The enhanced inhibition observed in coculture systems further suggests that microbial consortia provide a broader enzymatic repertoire, including glycosidases and esterases, which increase the extent of phenolic transformation and bioactivation. This results in a more diverse pool of bioactive compounds with complementary inhibitory mechanisms, including competitive and mixed-type inhibition, as reported for plant-derived polyphenols [29,31].

Overall, these results indicate that lactic fermentation enhances α-amylase inhibitory activity through the combined effects of phenolic release, structural modification, and increased molecular accessibility. The EP fraction provides a more potent inhibitory response due to the presence of readily diffusible, low-molecular-weight phenolics. In contrast, the HP fraction contributes additional inhibitory capacity following fermentation-induced depolymerization and release. The superior performance observed under coculture conditions highlights the importance of microbial interactions in maximizing the functional potential of phenolic compounds.

#### 3.3.2. α-Glucosidase Inhibition Activity

α-Glucosidase catalyzes the final step of carbohydrate digestion by hydrolyzing α-(1 → 4) and α-(1 → 6) glycosidic bonds in oligosaccharides at the intestinal brush border, thereby releasing glucose and increasing postprandial glycemia. Inhibition of this enzyme is therefore a well-established strategy to delay glucose absorption and attenuate glycemic excursions, contributing to the prevention and management of type 2 diabetes [32,33].

**Figure 5:**
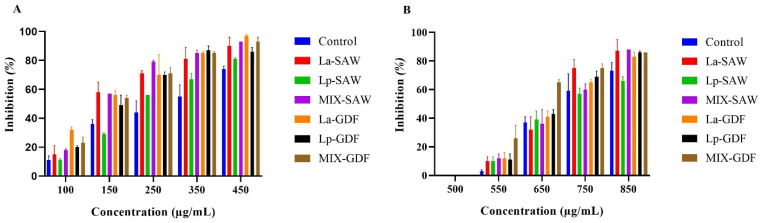
Enzymatic inhibition of α-glucosidase by phenolic extracts from fermented murta juice. (**A**) Extractable phenolic (EP) fraction; (**B**) hydrolyzable phenolic (HP) fraction. Results are expressed as percentage inhibition at different concentrations. La: *Lactobacillus acidophilus*; Lp: *Lactiplantibacillus plantarum*; MIX: 1:1 mixed culture; SAW: Simple Additive Weighting; GDF: Global Desirability Function.

As shown in Figure 5, both EP (A) and HP (B) fractions exhibited concentration-dependent inhibition of α-glucosidase, with progressively reduced activity at lower extract concentrations. Notably, all fermented EP extracts significantly outperformed the unfermented control (Figure 5A). Among them, La-SAW and MIX-SAW showed the highest inhibitory activity, reaching values close to 90% at 450 µg/mL and maintaining substantial inhibition at intermediate concentrations (250–300 µg/mL). This enhanced activity can be mechanistically attributed to fermentation-induced transformations of phenolic compounds, including deglycosylation, de-esterification, and partial depolymerization, which generate smaller, more bioaccessible molecules with increased affinity for the enzyme active site.

At the molecular level, polyphenols inhibit α-glucosidase through a combination of competitive and mixed-type mechanisms, involving interactions with key catalytic residues such as Asp518 and Asp616, as well as with peripheral binding sites that modulate enzyme conformation [34,35]. Hydrogen bonding between hydroxyl groups of phenolics and amino acid residues, together with π–π stacking interactions with aromatic residues, contributes to the stabilization of enzyme–inhibitor complexes. Structural features such as hydroxylation pattern, degree of conjugation, and absence of glycosylation enhance inhibitory potency by improving molecular planarity and hydrophobic interactions with the active site. These structure–activity relationships are consistent with the superior performance of fermented EP fractions, in which microbial metabolism promotes the formation of aglycones and other low-molecular-weight derivatives with higher inhibitory activity.

In addition to direct enzyme binding, phenolic compounds may exert indirect inhibitory effects by altering the enzyme’s microenvironment, including local pH and redox conditions, thereby affecting catalytic activity. Fermentation may further enhance these effects by increasing the diversity and availability of phenolic metabolites with different enzyme-binding capacities [23]. The high inhibition levels observed are consistent with previous reports on fermented plant matrices, including pumpkin-based beverages and murta juice, where fermentation significantly improved α-glucosidase inhibitory activity [13,36].

The HP fraction (Figure 5B) also exhibited strong α-glucosidase inhibition, with a clear concentration-dependent response. At the highest concentration, most fermented extracts achieved inhibition levels above 80%, markedly higher than those of the unfermented control. This behavior indicates that hydrolyzable polyphenols, including tannins and phenolic polymers, contribute significantly to enzyme inhibition. Unlike low-molecular-weight phenolics, these compounds may interact with α-glucosidase at multiple binding sites, leading to non-competitive or mixed-type inhibition. Their larger molecular size and multiple hydroxyl groups enable extensive hydrogen bonding and potential conformational changes in the enzyme, thereby reducing catalytic efficiency.

The enhanced activity of HP fractions following fermentation suggests that microbial enzymatic processes, including tannase and esterase activities, promote the partial de-polymerization and release of bound phenolics, increasing their accessibility and reactivity. This is consistent with previous studies reporting potent α-glucosidase inhibition by hydrolyzable tannin-rich fractions from *Eugenia* species and other plant matrices [37,38]. Similarly, phenolic polymers and complex fractions have been shown to exhibit strong inhibitory effects due to multivalent interactions with the enzyme surface [39].

Overall, these results demonstrate that lactic fermentation enhances α-glucosidase inhibitory activity through the combined effects of phenolic release, structural transformation, and increased molecular accessibility. EP fractions exhibit a highly potent inhibitory response, driven by low-molecular-weight phenolics with strong affinity for the enzyme’s active site. In contrast, HP fractions contribute complementary inhibition through multivalent interactions and progressive release of bioactive compounds. The superior performance observed under coculture conditions highlights the role of microbial interactions in maximizing the functional potential of phenolic compounds. It underscores the importance of considering both extractable and hydrolyzable fractions in evaluating glycemic regulatory properties.

#### 3.3.3. DPP-IV Inhibitory Activity

Dipeptidyl peptidase IV (DPP-IV) is a serine protease responsible for the rapid degradation of incretin hormones, particularly glucagon-like peptide-1 (GLP-1) and glucose-dependent insulinotropic polypeptide (GIP), which play a central role in glucose homeostasis by stimulating insulin secretion and suppressing glucagon release. Inhibition of DPP-IV prolongs incretin activity, thereby improving glycemic control and representing a validated therapeutic strategy for the management of type 2 diabetes [40,41].

**Figure 6:**
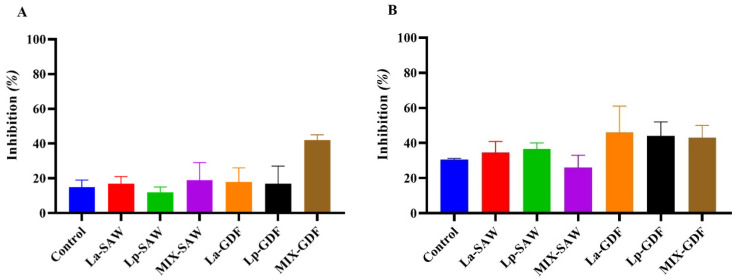
DPP-IV inhibitory activity of different phenolic fractions obtained from fermented murta juice. (**A**) Extractable phenolic (EP) fraction; (**B**) hydrolyzable phenolic (HP) fraction. Results are expressed as percentage inhibition. La: *Lactobacillus acidophilus*; Lp: *Lactiplantibacillus plantarum*; MIX: 1:1 mixed culture; SAW: Simple Additive Weighting; GDF: Global Desirability Function.

As shown in Figure 6, both EP (A) and HP (B) fractions exhibited inhibition of DPP-IV at a concentration of 1 mg/mL.

Regarding the EP fractions, only MIX-GDF showed significantly higher DPP-IV inhibitory activity than the unfermented control, reaching approximately 40%. This result suggests that coculture fermentation enhanced the release or formation of bioactive compounds, particularly phenolic derivatives and microbial metabolites, contributing to enzyme inhibition. In the HP fractions, La-GDF, Lp-GDF, and MIX-GDF exhibited increased inhibitory activity, with inhibition levels around 40%. Overall, HP fractions showed higher DPP-IV inhibitory activity than EP fractions, which may be associated with differences in phenolic composition and the greater contribution of bound phenolic compounds. The enhanced inhibition observed in fermented HP fractions suggests that microbial enzymatic reactions, particularly β-glucosidase-mediated hydrolysis and bio-transformation, may promote the release of smaller phenolic compounds and other bioac-tive metabolites capable of interacting with DPP-IV [42]. In addition, polymeric phenolics and tannins can inhibit the enzyme through multivalent interactions with the enzyme surface, potentially inducing conformational changes that reduce catalytic activity.

At the molecular level, DPP-IV inhibition by food-derived phenolics and flavonoids has been associated with non-covalent interactions within the enzyme’s binding pockets, particularly the S1 and S2 regions, involving key residues such as Ser630, Glu205, and Glu206, as supported by in vitro and molecular docking studies [41,43]. Inhibitory potency appears to depend on structural characteristics such as hydroxylation pattern, conjugation, glycosylation state, and molecular flexibility, which influence binding affinity and accessibility to the enzyme pocket [41,44]. These structure–activity relationships are consistent with the enhanced inhibition observed in fermented fractions, in which microbial metabolism promotes phenolic biotransformations, including the formation of aglycones and low-molecular-weight derivatives. Similarly, studies in fermented berry matrices have shown that lactic acid fermentation can increase bioactive phenolics, including gallic, chlorogenic, caffeic, and protocatechuic acids, as well as flavonoid derivatives, which may interact with DPP-IV through hydrogen bonding and hydrophobic interactions. Therefore, the improved inhibitory activity observed in fermented murta juice fractions may be partially associated with microbial-mediated modifications of the phenolic profile [42].

Overall, these results demonstrate that lactic acid fermentation enhances the antidiabetic potential of murta juice phenolic fractions by improving their inhibitory activity against α-amylase, α-glucosidase, and DPP-IV. This effect appears to be associated with microbial-mediated phenolic biotransformation, thereby increasing the availability of bioactive compounds with enzyme-inhibitory activity. The complementary contributions of the EP and HP fractions highlight the importance of considering both extractable and bound phenolics when evaluating the functional potential of fermented berry matrices.

### 3.4. Cytoprotective Effects: Cell Integrity and Intracellular ROS Modulation

ROS are essential signaling mediators in intestinal epithelial cells, where they participate in proliferation, differentiation, and redox-sensitive signaling. However, excessive ROS accumulation disrupts redox homeostasis, damages lipids, proteins, and DNA, impairs mitochondrial function, and can ultimately trigger apoptosis [45]. In this context, the effects of the EP and HP phenolic fractions of fermented murta juice on Caco-2 cell viability and on H_2_O_2_-induced oxidative stress were evaluated.

Cell viability assessment is a necessary first step to exclude nonspecific cytotoxicity before interpreting antioxidant or cytoprotective effects. Under the tested conditions, neither fraction, at concentrations of 45–225 μg/mL, reduced Caco-2 cell viability after 72 h of exposure, with values remaining close to 100% for all treatments. These results indicate that the phenolic fractions are well tolerated by intestinal epithelial cells within the evaluated range. However, in the H_2_O_2_-induced oxidative stress assay, cells exposed only to the oxidizing agent exhibited an almost complete loss of viability, confirming severe oxidative damage. In this context, the phenolic fractions exhibited marked antioxidant activity in the intracellular ROS assay, significantly reducing the H_2_O_2_-induced signal, suggesting a protective effect against oxidative damage. This behavior is consistent with previous studies on berry-derived phenolic fractions in intestinal models, including murtilla pomace fractions, which likewise preserved Caco-2 metabolic activity while reducing oxidative stress [46]. To rule out potential fluorescence quenching from phenolic extracts, a cell-free fluorescence interference assay was performed by incubating DCFH-DA with each fraction at the highest tested concentration (225 μg/mL) and measuring the residual fluorescence under the same instrumental conditions. No significant quenching was observed (<5% signal reduction compared to the vehicle control), confirming that the intracellular ROS inhibition values reported below reflect genuine antioxidant activity rather than spectroscopic artifacts.

**Figure 7:**
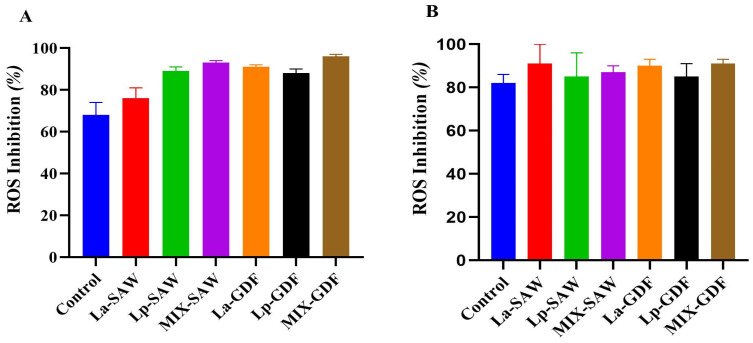
Intracellular ROS inhibition in Caco-2 cells by phenolic extracts obtained from fermented murta juice. (**A**) EP fraction; (**B**) HP fraction. Results are expressed as the percentage of ROS inhibition after oxidative stress induction with H_2_O_2_.

Intracellular antioxidant activity was then assessed using the DCFH-DA probe under H_2_O_2_ challenge. As shown in Figure 7A,B, several fermented fractions suppressed more than 90% of the relative fluorescence signal, indicating a marked attenuation of intracellular ROS accumulation. Among them, MIX-GDF and MIX-SAW showed the greatest activity, with near-complete (>95%) suppression of the oxidative signal. These results strongly suggest that fermentation enhanced the generation, release, or activation of compounds with intracellular antioxidant efficacy. Current evidence indicates that lactic fermentation can increase the functional potency of plant phenolics through deglycosylation, de-esterification, depolymerization, and related biotransformations that yield smaller phenolic acids, aglycones, and other derivatives with improved bioaccessibility and stronger interactions with cellular redox systems. Fermentation has also been reported to enrich fruit and vegetable juices in bioactive compounds and to improve overall phenolic bioactivity [47].

Mechanistically, the marked reduction in intracellular ROS is unlikely to be explained only by direct radical scavenging. In intestinal cells, polyphenols frequently act through a combination of direct and indirect antioxidant mechanisms. Directly, compounds such as phenolic acids, flavonols, and anthocyanin-derived metabolites can neutralize ROS or intercept radical chain reactions. Indirectly, and often more importantly at physiologically relevant concentrations, they modulate endogenous defense pathways, particularly the Keap1–Nrf2 axis, thereby increasing the expression of phase II and antioxidant enzymes, such as heme oxygenase-1 and glutathione-related enzymes, and other cytoprotective systems. Recent reviews on intestinal redox biology have emphasized that polyphenols may undergo oxidation to electrophilic quinone-like intermediates that can modify thiol groups in the Keap1–Nrf2 complex, triggering an adaptive antioxidant response rather than acting solely as stoichiometric scavengers [48]. This interpretation is fully compatible with the strong intracellular protection we observed after juice fermentation.

The superior performance of the coculture-fermented fractions further suggests that microbial interactions enhanced the biochemical diversity of the resulting phenolic pool. Cocultures typically provide a broader repertoire of glycosidases, esterases, decarboxylases, and reductases, thereby increasing the probability of producing metabolites with improved cell permeability and higher reactivity toward intracellular redox targets. This may be particularly relevant for berry phenolics, whose native glycosylated and polymeric forms often have limited cellular accessibility, whereas their fermentation-derived metabolites can display stronger intracellular antioxidant effects. Similar trends have been reported for fermented blueberry juice, in which *Lactiplantibacillus plantarum* fermentation increased total phenolics and improved functional activity, including protection against oxidative stress in Caco-2 models. Related findings have also been reported for fermented berry systems, in which lactic fermentation increased levels of phenols, flavonoids, and anthocyanins and enhanced cellular antioxidant protection, as measured by DCFH-DA assays [49].

The present results also align with the broader berry literature in epithelial models. Anthocyanin-rich bilberry extracts have been shown to reduce intracellular ROS in Caco-2 cells, and berry phenolics more generally are recognized as potent modulators of intestinal oxidative status and barrier-associated signaling [50]. Although these studies were not always conducted with fermented juices, they support the concept that berry-derived phenolics are particularly effective in intestinal redox regulation and provide a mechanistic backdrop for the high activity of fermented murta fractions observed here.

The behavior of the HP fraction deserves particular attention. Even though hydrolyzable or matrix-associated phenolics are often less immediately accessible than soluble fractions, they can become highly active after release or transformation. Previous work on murtilla pomace and calafate byproducts showed that insoluble-bound phenolic fractions can exert substantial antioxidant protection in Caco-2 cells, in some cases outperforming free phenolic fractions, suggesting that bound phenolics should not be viewed as biologically inert reservoirs. Instead, once liberated by processing, hydrolysis, or fermentation, they can contribute decisively to intracellular protection. The strong activity of the fermented HP fractions in the present study is therefore mechanistically plausible and consistent with the emerging view that bound phenolics constitute an important source of delayed but functionally relevant antioxidant capacity [47].

In contrast, no relevant protection to cell viability under oxidative challenge was observed. This apparent discrepancy between intra- and extracellular behavior is consistent with the compartmentalized nature of cellular redox biology. ROS formation and detoxification occur in spatially distinct microdomains, including mitochondria, NADPH oxidase-associated membranes, the cytosol, and other organelle-associated compartments, and the biological consequences of antioxidant intervention depend strongly on where reactive species are generated and where antioxidants or their metabolites accumulate. Polyphenols that enter cells, interact with membranes, or activate intracellular signaling may therefore show pronounced effects in intracellular assays while exerting limited effects in extracellular environments where transport, localization, and target accessibility differ. Rather than weakening the antioxidant interpretation, the lack of extracellular protection reinforces the conclusion that the major activity of these fractions is intracellular redox modulation [49].

Overall, the results indicate that the polyphenolic fractions of fermented murta juice do not exhibit cytotoxicity in Caco-2 cells and exert strong intracellular antioxidant protection under oxidative stress, an effect that is enhanced after fermentation due to biotransformation into more bioactive and bioavailable compounds, consistent with reports from other fermented fruit and berry byproduct systems [50].

### 3.5. Phenolic–Bioactivity Connectivity Network: A Dual-Functional Architecture

**Figure 8:**
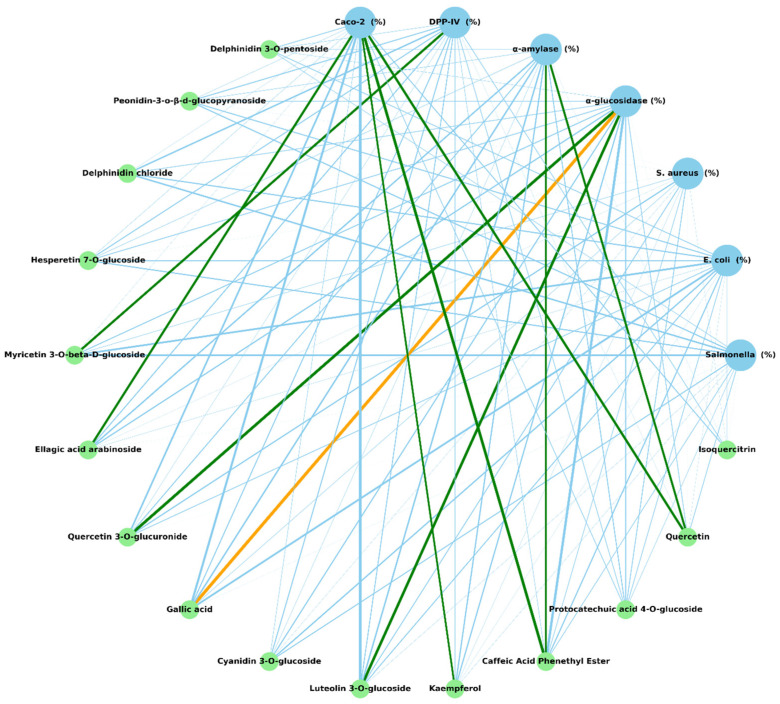
Correlation matrix of the Mantel test and network diagram between phenolic compounds and bioactivities. Green nodes represent individual phenolic compounds, while blue nodes represent the biological variables evaluated (enzymatic inhibitory activity, antimicrobial activity, and intracellular ROS inhibition). Lines indicate significant Mantel correlations: green represents significant associations (*p* < 0.05), orange represents highly significant associations (*p* < 0.01), and blue represents non-significant associations. Line thickness represents the magnitude of the Mantel correlation coefficient (r).

To integrate the three functional axes evaluated above, a bipartite correlation network (Figure 8) was constructed linking 15 phenolic compounds to antimicrobial, antidiabetic, and cellular antioxidant endpoints. Beyond summarizing pairwise associations, this representation captures the functional organization of fermented murta juice as a multicomponent system in which some bioactivities are associated with structurally defined phenolics. In contrast, others emerge from distributed contributions across the phenolic pool. This type of connectivity analysis is increasingly used in food science to relate compositional remodeling to functional outcomes. Likewise, correlation and relevance ana-lyses have been used on fermented fruit matrices to link changes in phenolic composition to antioxidant and functional responses. For example, in LAB-fermented hawthorn pulp, phenolic compounds were significantly correlated with antioxidant and enzyme-inhibitory activities, with metabolomic analysis linking these effects to phenolic biotransformation pathways [51]. Similarly, in fermented berry systems such as black chokeberry juice, Pearson correlation analysis demonstrated that fermentation-driven transformations of phenolic acids, flavonoids, and anthocyanins were directly associated with enhanced antioxidant activity [52]. Comparable relationships have also been reported in composite fruit fermentations, where specific phenolics showed positive correlations with antioxidant capacity [53], supporting the interpretation that fermentation-induced functional improvements can be understood through compound–bioactivity connectivity rather than solely through changes in concentration.

In wine research, correlation-based approaches, including network heat maps, have been applied to link total phenols, flavonoids and specific phenolic acids with antioxidant activity and sensory attributes. These analyses have shown that individual compounds do not always drive functional properties; rather, they often emerge from coordinated shifts in the overall phenolic composition, highlighting the relevance of network-level interpretation [54,55,56].

The network included Mantel correlation coefficients (r) along with two significance thresholds to distinguish between the magnitude and statistical significance of phenolic–bioactivity associations: green edges indicate significant correlations (*p* < 0.05), whereas orange edges indicate highly significant correlations (*p* < 0.01). Edge thickness represents the magnitude of the Mantel correlation coefficient (r). This combined approach was used to facilitate visual interpretation of both the strength and statistical relevance of phenolic–bioactivity associations. Given the large number of pairwise comparisons, *p*-values were interpreted cautiously and should be considered exploratory rather than confirmatory.

The first mode identified in the present network corresponds to specific phenolic–bioactivity associations, particularly those related to enzyme inhibition and cellular protection. For α-amylase inhibition, positive Mantel correlations were observed with quercetin (r = 0.72, *p* = 0.02) and caffeic acid phenethyl ester (r = 0.68, *p* = 0.03), compounds previously described as potent enzyme-binding flavonoids or phenolic derivatives, owing to their hydroxylation patterns and aromatic rings that favor hydrogen bonding and hydrophobic interactions within the catalytic pocket. For α-glucosidase inhibition, the strongest associations involved gallic acid (r = 0.87, *p* = 0.01), quercetin-3-O-glucuronide (r = 0.79, *p* = 0.02), and luteolin-3-O-glucoside (r = 0.76, *p* = 0.03), in agreement with the well-established structure dependence of flavonoid-mediated inhibition. In the same way, myricetin-3-O-beta-D-glucoside was associated with DPP-IV inhibition (r = 0.67, *p* = 0.03), supporting the contribution of myricetin derivatives to this activity. Significant associations between ellagic acid arabinoside (r = 0.76, *p* = 0.03), caffeic acid phenethyl ester (r = 0.78, *p* = 0.04), quercetin (r = 0.79, *p* = 0.01), and the Caco-2 antioxidant response were also observed, supporting their potential contribution to intracellular ROS modulation and cytoprotective signaling.

The second mode corresponds to combined or network-driven effects, which are particularly evident for antimicrobial activity. In contrast to the digestive-enzyme and cell-based endpoints, the antimicrobial responses against *S. enterica*, *E. coli*, and *S. aureus* were not explained by a single dominant node but rather by weaker, more diffuse associations distributed across several phenolics. This interpretation is consistent with the broader food literature, in which antimicrobial activity is often described as an emergent property of mixtures rather than as the direct consequence of a single major phenolic compound. In fermented blueberry juice, for example, lactic fermentation enhanced phenolic content and antioxidant capacity while also producing correlated shifts in organic acids and phenolic profiles, supporting the idea that functional outputs in fermented berry systems result from coordinated metabolic remodeling rather than from isolated compound changes. Similarly, correlation-based analyses of fermented rose products and polyphenol-enriched wines have shown that certain endpoints map well onto the overall phenolic architecture. In contrast, others are less well explained by single compounds and instead reflect collective changes in the matrix. This framework is particularly relevant to antimicrobial effects because bacterial sensitivity depends not only on phenolic structure but also on membrane composition, outer-envelope architecture, and the potential for additive or partially synergistic interactions among multiple compounds. Thus, the heterogeneous antimicrobial connectivity observed here is not a weakness of the model, but rather a biologically coherent signature of mixed, species-dependent phenolic action [57].

This distinction between specific and distributed connectivity is important because it provides a mechanistic interpretation of fermentation-driven phenolic remodeling. In matrices where fermentation primarily increases the abundance of compounds with known enzyme-binding motifs, one expects more localized phenolic–bioactivity associations, as observed here for α-amylase, α-glucosidase, DPP-IV, and modulation of intracellular ROS. By contrast, when fermentation alters the matrix more globally through deglycosylation, de-esterification, partial depolymerization [3], and concomitant changes in acids and other metabolites, the resulting activity may be better represented as a network property.

Taken together, the phenolic-bioactivity network integrates antimicrobial, antidiabetic, and intracellular antioxidant actions into a unified functional framework. The results reveal a dual-function architecture in which structurally defined flavonoids and related phenolic compounds are associated with specific bioactivities. At the same time, the collective interactions of phenols appear to govern broader responses, such as antimicrobial inhibition. This behavior is consistent with evidence that phenolic compounds exhibit multiple biological activities and act on various cellular and molecular processes, including oxidative stress, inflammation, and glucose metabolism [58]. In this sense, the network provides a rational basis for designing targeted fermentation strategies to shift phenolic connectivity toward desired functional profiles, rather than relying solely on empirical screening [50].

## 4. Conclusions

This study demonstrates that controlled lactic fermentation restructures the phenolic composition of murta juice in both EP and HP fractions, resulting in measurable changes in antimicrobial, enzyme-inhibitory, and cytoprotective activity. Coculture fermentation (MIX-GDF) generated the most compositionally distinct phenolic profile and was associated with enhanced performance across multiple bioactivity axes.

EP fractions contributed more strongly to antimicrobial activity and α-glucosidase inhibition, whereas HP fractions tended to show greater involvement in DPP-IV inhibition and intracellular ROS reduction. Neither fraction exhibited cytotoxicity toward Caco-2 cells at the concentrations tested, supporting their potential as safe functional ingredients. The integration of compositional data with a bipartite phenolic–bioactivity network revealed a dual-functional architecture: specific flavonoid–enzyme associations were linked to enzyme inhibition, whereas more diffuse collective interactions appeared to contribute to antimicrobial responses. This connectivity framework offers a transferable analytical approach for linking composition to function in fermented plant matrices. It provides a basis for the rational design of fermentation strategies targeting defined bioactivity profiles.

Future work should extend this approach to dose–response characterization of DPP-IV inhibition, statistical comparison of EP and HP fractions across functional endpoints, in vivo validation of the bioactivities reported here, and application of the connectivity framework to other berry-based fermented systems.

## Figures and Tables

**Table 1:** Optimum fermentation conditions of murta juice obtained using Simple Additive Weighting (SAW) and Global Desirability Function (GDF) optimization strategies. La: *Lactobacillus acidophilus*; Lp: *Lactiplantibacillus plantarum*; MIX: 1:1 mixed culture of both strains.

Treatment	La-SAW	Lp-SAW	MIX-SAW	La-GDF	Lp-GDF	MIX-GDF
T (°C)	36.0	35.0	35.0	39.0	39.0	38.0
pH	6.0	6.0	6.0	6.0	6.0	6.0
Inoculum (%)	2.1	3.6	9.2	10.0	10.0	10.0

## Data Availability

The experimental data supporting the findings of this study are available from the corresponding author upon reasonable request.
